# *Dioscorea* Plants: A Genus Rich in Vital Nutra-pharmaceuticals-A Review

**DOI:** 10.22037/ijpr.2019.112501.13795

**Published:** 2019

**Authors:** Bahare Salehi, Bilge Sener, Mehtap Kilic, Javad Sharifi-Rad, Rabia Naz, Zubaida Yousaf, Fhatuwani Nixwell Mudau, Patrick Valere Tsouh Fokou, Shahira M. Ezzat, Mahitab H. El Bishbishy, Yasaman Taheri, Giuseppe Lucariello, Alessandra Durazzo, Massimo Lucarini, Hafiz Ansar Rasul Suleria, Antonello Santini

**Affiliations:** a *Student Research Committee, School of Medicine, Bam University of Medical Sciences, Bam, Iran. *; b *Department of Pharmacognosy, Faculty of Pharmacy, Gazi University, 06330 Ankara, Turkey.*; c *Zabol Medicinal Plants Research Center, Zabol University of Medical Sciences, Zabol, Iran. *; d *Department of Biosciences, COMSATS University, Park Road, Islamabad, Pakistan.*; e *Department of Botany, Lahore College for Women University, Jail Road Lahore, Pakistan. *; f *Department of Agriculture and Animal Health, College of Agriculture and Environmental Sciences, University of South Africa, Private Bag X6, Florida, 1710, South Africa. *; g *gDepartment of Biochemistry, Faculty of Science, University of Yaounde 1, Yaounde Po.Box 812, Cameroon*; h *Department of Pharmacognosy, Faculty of Pharmacy, Cairo University, Kasr El-Ainy Street, Cairo 11562, Egypt. *; i *Department of Pharmacognosy, Faculty of Pharmacy, October University for Modern Sciences and Arts (MSA), 12611 Cairo, Egypt. *; j *Phytochemistry Research Center, Shahid Beheshti University of Medical Sciences, Tehran, Iran. *; k *Department of Pharmacology and Toxicology, School of Pharmacy, Shahid Beheshti University of Medical Sciences, Tehran, Iran.*; l *Department of Pharmacy, University of Napoli Federico II, Via D. Montesano 49, 80131 Napoli, Italy. *; m *CREA-Research Centre for Food and Nutrition, Via Ardeatina 546, 00178 Rome, Italy. *; n *Department of Agriculture and Food Systems, The University of Melbourne, Melbourne 3010, Australia.*

## Abstract

*Dioscorea* species, known as “Yams,” belong to family *Dioscoreaceae*. This genus consists of more than 600 species distributed from Africa, Asia, the Caribbean’s South America, and the South Pacific islands. Their organoleptic properties make them the most widely used carbohydrate food and dietary supplements. The underground and/or aerial tubers represent valuable sources of proteins, fats, and vitamins for millions of people in West Africa. This review gives a shot of secondary metabolites of *Dioscorea* plants, including steroids, clerodane diterpenes, quinones, cyanidins, phenolics, diarylheptanoids, and nitrogen-containing compounds. This review collected the evidence on biological properties of description *Dioscorea*, including *in-vitro *and *in-vivo *studies. *Dioscorea* species contain promising bioactive molecules *i.e.* diosgenin that support their different biological properties, including antioxidant, hypoglycaemic, hypolipidemic, anti- antimicrobial, inflammatory, antiproliferative, androgenic, estrogenic, and contraceptive drugs. Indeed, besides its nutrient values, *Dioscorea* is a potential source of bioactive substances of interest in the prevention/treatment of several diseases, and thus represents a great challenge in developing countries. However, ethnomedicinal potential should be validated and further researches on pharmacological properties and phytochemical composition should be carried out. Particularly, doing some studies to convert the preclinical results to clinical efficacy should be guaranteed.

Dioscorea, Food plant, Traditional use, Phytochemistry, Pharmacological activities

## Introduction

*Dioscorea* belongs to the family *Dioscoreaceae*, sub-family Dioscoreoideae, consisting of 600 species. *Dioscorea* species are native to the old-world including high temperatures tropics and subtropic regions of the world. The major part of species occur in West Africa, Southern Asia, and Tropical America: they are herbaceous climbers with rhizome or tubers *Dioscorea* species and, being usedin the formulation of pharmaceutical products, have a high medicinal, industrial and commercial importance (1). Indeed, *Dioscorea *species account for the most important dietary supplements ingredients used in cosmetics and pharmaceutical industries. It has also been commonly used by local people, mostly those who are engaged in the trade of medical plants worldwide. Indeed, tubers of different *Dioscorea *species are used as a cure for different diseases and ailments (cough, cold, stomach ache, leprosy, burns, fungal infections, dysentery, skin diseases, rheumatism, arthritis, *etc.*) in several formulations, and even for birth control (1). It is well known the potential of this plant is related due to the different phytochemical compounds found in *Dioscorea*. Tubers and roots contain steroidal sapogenins, mostly diosgenin as well as volatile compounds (2, 3). Chemical substances like diosgenin found in *Dioscorea *are commercially used in the pharmaceutical industry. The high demand for the medicinal use of this plant in different parts of the world suggests a strong need for conservation strategies. However, the conservation might not be easy or simple as it must involve plant protection and well-controlled access to its resources. There still a huge need for more research and information on the food, nutraceutical, and medicinal value worldwide of this plant species. Therefore, this review tends to fill this gap by summarizing data on the current medicinal importance of *Dioscorea* species.

***Dioscorea*** **plants phytochemical composition**

**Figure 1:**
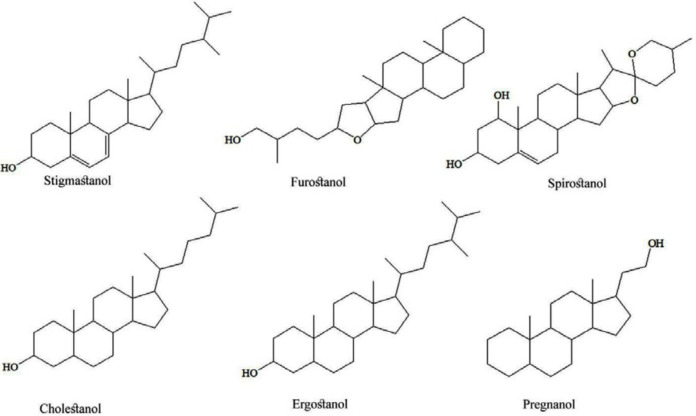
Chemical structures of selected compounds isolated from *Dioscorea* species

Researches have been brought new knowledge about chemical compounds in tubers of *Dioscorea* species and possible medicinal usage. The most common secondary metabolites are saponins, and more than 100 steroidal saponins (based on aglycon part as stigmastanol, furostanol, spirostanol, cholestanol, ergostanol, and pregnanol glycosides (Figure 1)) were isolated from various *Dioscorea *species. Besides these steroids, clerodane diterpenes, phenolics, cyanidins, quinones, diarylheptanoids, and nitrogen-containing compounds in the tubers of *Dioscorea *species were quantified (4). Steroidal saponins mainly consist of furostane, a pentacyclic ring system with a sixth open ring; spirostane, a hexacyclic ring system; and pregnane, a tetracyclic ring system. The sugar part is mainly composed of glucose and rhamnose in various proportions and linkages. Steroidal saponins are glycosides consisting of an aglycone (diosgenin) and several glycosyl moieties. The most common sugars encountered in saponins are pentoses (arabinose, xylose, *etc.*), hexoses (glucose, galactose, *etc.*) and 6-deoxyhexose (rhamnose, *etc.*). The saponins of this plant species are both water-soluble and water-insoluble steroid saponins. *Dioscorea *species are well known for containing steroidal saponins, which were used as marker compounds for quality control of the botanical products. As reported by Jesus *et al.*, 2016, diosgenin (3-*β*-hydroxy-5-spirostene) is the primary furostanol saponin found in several plants, including *Dioscorea* species, and is described as a promising bioactive compound with several medicinal properties, *i.e.* hypolipidemic, antioxidant, anti-inflammatory, hypoglycaemic, and antiproliferative activities (5). Diosgenin obtained by hydrolysis of yam saponin were the main raw material for the industrial production of steroid drug *i.e.* anti-inflammatory, androgenic, estrogenic, and contraceptive drugs, by underlying its potential in the prevention/treatment of several diseases. *Dioscorea villosa* L. roots and rhizomes, also are known as “wild yam”, is a rich source of diosgenin. Today, dietary supplements containing wild yam extracts are popular among women for the alleviation of menopausal symptoms and are widely used as alternatives to hormone replacement therapy (5).

*Dioscorea alata L.*

*Dioscorea alata* L. is one of the most popular varieties of yams. *Dioscorea alata (D. alata) *were implicated in the promotion of the health of postmenopausal women. Wild Mexican yam was marketed for the treatment of irritability, hot flashes, insomnia, and depression. Cheng *et al.*, 2007, reported the isolation and identification of two new compounds, hydro-Q_9_ chromene, and *γ*-tocopherol-9; together with four known compounds (*RRR*-*α*-tocopherol, coenzyme Q_9_, cycloartane, and 1-feruloylglycerol): these compounds had phenolic hydroxyl group in common with the known phytoestrogens and have also antioxidant activity. Moreover, their estrogenic activity was evaluated based on the ligand-dependent transcription activation assay (6). 

*Dioscorea antaly Jum. and H. Perrier*

*Dioscorea antaly* Jum. and H. Perrier is originated from Madagascar, diffused in the West and North-West regions. The study of Rakatobe *et al.,* 2010 reported Two clerodane diterpenoids, antadiosbulbins A and B and two furanoid 19-norclerodane diterpenes, 8-epidiosbulbins E and G along with the known diterpenoid diosbulbin E as well as nine known phenolics including five phenanthrenes and four flavonoids in the ethyl acetate soluble part of the methanolic extract of the tubers (7).

*Dioscorea bulbifera L.*

*Dioscorea bulbifera L. (D. bulbifera L.)*, commonly known as air potato, is originated from Africa and Southern Asia. The rhizome of *D. bulbifera* L. is called as “Huang Yao Zi” in traditional Chinese medicine and used for the treatment of thyroid diseases and cancers. In the northern districts of Bangladesh, this herb is used for the treatment of cancers and leprosy. This plant has two types of storage organs, namely bulbils in the leaf axils of the stem and tubers. Bulbils have been used as a folk remedy to treat diarrhea, conjunctivitis, and the extracts of the plant exhibit anti-tumor activity. The mannose-binding lectin from bulbils of *D. bulbifera* was studied for its clinical potential in HIV and cancer research (8). Phytochemical studies reported as main bioactive compounds of this plant: flavonoids, clerodane diterpenoids, and steroidal saponins and phenolic compounds. As instance, Liu *et al.* 2011 reported the isolation and identification of two new compounds, bibenzyl type-2,5,2′,5′-tetrahydroxy-3′-methoxybibenzyl and diarylheptanone containing a dihydrophenanthrene moiety named as diobulbinone A along with sixteen known compounds (9). Nine norclerodane diterpenoids were isolated from the tubers of *D. bulbifera *L. including two new compounds (diosbulbin N and diosbulbin P), and a naturally occurring compound (diosbulbin O) along with six known diosbulbins A-D, F, and G (10). Another study also showed the molecular changes of liver dysfunction and reveal overall metabolic and physiological mechanisms of the subchronic toxic effect of *Dioscorea bulbifera* rhizome (11).

*Dioscorea cayenensis Lam.-Holl*

*Dioscorea cayenensis* Lam.-Holl known as “yellow yam” is native to tropical West Africa and its rhizomes are used as food and as a remedy for treating burns and fevers. A new furostanol glycoside namely 26-*O*-*β* -D-glucopyranosyl-3*β*,26-dihydroxy-20,22- seco-25(*R*)-furost-5-en-20,22-dione-3-*O*-*α*-L-rhamnopyranosyl-(1→4)-*α*-L-rhamnopyranosyl-(1→4)-[*α*-L-rhamnopyranosyl-(1→2)]-*β*-D-glucopyranoside was isolated from the methanolic extract of the rhizome of *Dioscorea cayenensis *growing in Cameroon, together with the known spirostanol saponins described as methyl protodioscin, asperoside and prosapogenin A of dioscin (12). Prosapogenin A of dioscin having a spirostan skeleton showed antifungal activity against *Candida *species with MIC values between 6.25 and 25 mg/mL (12). Two new fatty acid-spirostan steroid glycoside esters, progenin III palmitate, and progenin III linoleate, were isolated from the methanol extract of the rhizomes of *Dioscorea cayenensis* (13). It is worth mentioning the recent studies on the nutrient and antinutrient composition of yellow yam (*Dioscorea cayenensis*) and their products; as instance raw *Dioscorea cayenensis* tubers contained 66.79 g moisture, 2.62 g crude protein, 0.27 g lipid, 0.17 g fiber, 0.63 g ash, 29.69 g carbohydrates, 262.30 mg potassium, 61.53 mg magnesium, 0.79 mg iron, 0.39 mg zinc, and yielded 108.26 kcal energy with insignificant vitamin content/100 g edible portion (14).

*Dioscorea esculenta (Lour.) Burk.*

*Dioscorea esculenta* also called as a “lesser yam” of *Dioscorea* species, is widely distributed Southern Asia and the Pacific. The tubers of *D. esculenta* were used traditionally in the treatment of various diseases. Moreover, fiteen steroidal saponins were isolated from its tuber powder, and from the leaf of *Dioscorea esculenta (D. esculenta)*; four of them belonged to furostanol-type saponin,eleven as spirostane-type saponins (15).

*Dioscorea japonica Thunb.*

The rhizome of *Dioscorea japonica* Thunb., known as “San Yak” in Korea, is used as food and medicine. The plant was used in traditional Chinese medicine to control/eliminate diarrhea, dilute sputum, improve anorexia, and moisturize skin (16). Phytochemical studies on *Dioscorea japonica (D. japonica) *showed the presence of active hypoglycemic compounds (dioscorans A–F), sesquiterpene, and acetophenone along with the steroidal constituents, including spirostane, furostane, and cholestane types (16).

*Dioscorea membranacea Pierre*

The rhizomes of *Dioscorea membrancea* (*D. membranacea*) Pierre have been used in Thai traditional medicine. As instance, Thongdeeying *et al.* 2016 isolated from the cytotoxic chloroform fraction of the rhizomes a new steroid (epi-panthogenin B), a known steroid (panthogenin B), two napthofuranoxepins dioscorealide A and dioscorealide B, phenanthraquinone (dioscoreanone) and three phenanthrene; the same authors reported that also, three steroids (*β*-sitosterol, stigmasterol, and *β*-D-sitosterolglucoside) and two steroidal saponins [(diosgenin-3-*O*-*α*-L-rhamnopyranosyl (1→2)-*β*-D-glucopyranoside and diosgenin-3-*O*-*β*-D-glucopyranosyl(1-3)-*β*-D-glucopyranoside)] were obtained from *D. membranacea* (17).

*Dioscorea nipponica Makino*

*Dioscorea nipponica (D. nipponica) *Makino is bitter-sweet in taste and warm in nature according to the traditional Chinese medicine. The rhizomes of *D. nipponica *were traditionally used in China as herbal medicine and food supplement for more than four thousand years; particularly, these plant species mainly act on the liver, kidney, and lungs, displaying anti-rheumatic, analgesic, blood circulation-stimulating, lung channel-dredging, digestive, anti-diuretic, anti-tussive, panting-calming, and phlegm-dispelling activities. Medicinally, it is commonly used for the treatment of rheumatoid arthritis, pain in the legs and lumbar area, Kashin-Beck disease, bruises, sprains, chronic bronchitis, cough and asthma and communication, and adherence to therapy is one of the main concerns (4, 18 and 19). Recently, it has been used as an important industrial raw material for the synthesis of steroidal drugs. Concerning the phytochemical profile, twelve cyclic diarylheptanoids were isolated from the rhizomes of *Dioscorea nipponica* (20), among which two new cyclic diarylheptanoids, diosniponol A and B; moreover, as reported by the same authors, these compounds were evaluated for their effects on nitric oxide production without cell toxicity in lipopolysaccharide-activated BV-2 cells. Therefore, diarylheptanoid derivatives from *D. nipponica* may be potential candidates for the treatment of various neurodegenerative diseases associated with neuroinflammation (20). Another study described the water-soluble non-saponin components [benzyl 1-*O*-*β*-D-glucopyranoside, leonuriside A, icariside D2, pyrocatechol-1*-O*-*β*-D-glucopyranoside, (+) syringaresinol-4-*O*-*β*-D-glucopyranoside, cyclo-(leu-tyr), and adenosine], and phenolic acid (piscidic acid) from the rhizomes of *D. nipponica *(21). On the other hand, 7-oxodioscin a new spirostan-type steroid along with known dioseptemloside G, (25*R*)-dracaenoside G, orbiculatoside B, dioscin, progenin III, gracillin were determined. The presence of (3*β*,22*α*,25*R*)-26-(*β*-Dglucopyranosyloxy)-22-methoxyfurost-5-en-3-yl-*O*-[*α*-L -rhamnopyranosyl-(1→4)]-*β*-D-glucopyranoside and methylprotodioscin were also confirmed as furastan-type steroids (21). It is worth mentioning the study of Li *et al.* 2017 that explore the chemical basis of the rhizomes and aerial parts of *Dioscorea nipponica* Makino by a combination of cheminformatics and bioinformatics, particularly their potential therapeutic and toxicity targets were screened through the DrugBank’s or T3DB’s ChemQuery structure search: the compounds in the rhizomes possessed 391 potential therapeutic targets and 216 potential toxicity targets (22).

*Dioscorea opposita Thunb.*

*Dioscorea opposita* Thunb. has been cultivated in China, Japan, and Korea as a food, and widely used as traditional medicine, for a very long time. There are about 80 yam cultivars that are originated in China, including the so-called “trueborn” Chinese yam. The important ingredient of the yam is sugar, which provides energy and sweetness and adds to the general flavor of foods. Also, the Chinese yam assists in food digestion and is beneficial in cases of stomach disorders (23). Phytochemical investigations of *Dioscorea opposite (D. opposita) *have revealed many chemical components such as purine derivatives, phenanthrenes, stilbenes, sapogenins, and saponins. Phytochemical studies of the chloroform soluble fraction of *D. opposita* Thunb. have resulted in the isolation of four new compounds, two dihydrostilbenes: 3,5-dihydroxy-4- methoxybibenzyl, 3,3′,5- trihydroxy-2′-methoxybibenzyl; and dibenz-oxepins 10,11-dihydro-dibenz[b,f]oxepin-2,4-diol and 10,11-dihydro-4-methoxy-dibenz[b,f]oxepin-2-ol, together with an additional fifteen known compounds. All the nineteen isolated compounds were tested in the DPPH, superoxide anion radical scavenging assays and cyclooxygenases (COXs) inhibition assay. Among them, 3,3′,5-trihydroxy-2′-methoxybibenzyl showed the most potent COX-2 inhibitory activity (23). Bioassay-guided fractionation of *D. opposita *extracts led to the isolation and identification of 23 phenolic compounds. Of them, 15 compounds reduced porcine pancreatic lipase activity at IC_50_ values of less than 50 mM and from them 3,3′,5-trihydroxy-2′-methoxybibenzyl and (4*E*,6*E*)-1,7-bis(4-hydroxyphenyl)-4,6-heptadien-3-one showed the highest inhibition with an IC_50_ value of 8.8 mM and dose-dependently in the concentration range 5-100 mM (24). These findings provide that phenolic compounds with stilbenoids are considered to play important roles in the lipase inhibition of the *D. opposita *extract. Especially, the stilbenoids possessing 3,5-dihydroxybibenzyl moiety showed higher inhibitory potencies than the others. The lipase inhibitory activities of stilbenoids depend on the presence of the hydroxyl group in the C-3 position. The structural difference also influences the inhibitory effect of diarylheptanoids on pancreatic lipase. Dihydrostilbene phenanthrene and diarylheptanoid structures were deemed to be responsible for lipase inhibition activity of this species (24). *D. opposita* Thunb. is also rich in starch, water-soluble polysaccharides, and mucilage defined as a polysaccharide with unique viscosity characteristics are widely used in the pharmaceutical and food industries as a thickening agent and emulsion stabilizer. These agents are important in the food industry as they improve the sensory quality, flavor, texture, palatability, mouthfeel, and general appearance of the final products. Therefore, the mucilage of this plant species is a potential candidate for food emulsifier resource of a natural emulsifier, especially under alkaline conditions obtained from industrial processing waste such as Gum Arabic, Yellow mustard, and Chia (*Salvia hispanica* L.) (25).

*Dioscorea polygonoides Humb. et Bonpl.*

*D. polygonoides *Humb. et Bonpl. is distributed from Mexico to Brazil, including Colombia. The phytochemical investigation of the tubers of *D. polygonoides *has resulted in the isolation of three new polyhydroxylated spirostanol saponins determined as (23*S*,24*R*,25*S*)-23,24-dihydroxyspirost-5-en-3*β*-yl-*O*-*α*-L-rhamnopyranosyl-(1→2)-*β*-D-glucopyranosid, (23*S*,25*R*)-12*α*,17*α*,23-trihydroxyspirost-5-en-3*β*-yl-*O*-*α*-L-rhamnopyranosyl-(1→2)-*β* -D-glucopyranoside, and (23*S*,25*R*)-14*α*,17*α*,23-trihydroxyspirost-5-en-3*β*-yl-*O*-*α*-L-rhamnopyranosyl-(1→2)-*β*-D-glucopyrano-side (26).

*Dioscorea preussii Pax*

*D. preussii* Pax collected in Bambui, Cameroon, was reported to have *in-vitro *cytotoxic, antileishmanial and antifungal activities. Three new steroidal saponins, named as diospreussinosides A, B, C, along with two known compounds were determined as (25*R*)-17*α-*hydroxyspirost-5-en-3*β*-yl *O*-*α*-L-rhamnopyranosyl-(1→4)*-O*-*α*-L-rhamnopyranosyl-(1 →4)-*β*-D-glucopyranoside and (25*R*)-17*α*-hydroxyspirost-5-en-3*β*-yl *O*-*α*-L-rhamnopyranosyl-(1 →2)-*O*-[*O*-*α*-L-rhamnopyranosyl-(1→4)-*α*-L-rhamnopyranosyl-(1→4)]-*β*-D-glucopyranoside were isolated from rhizomes of *D. preussii* (27).

*Dioscorea pyrifolia Kunth*

*D. pyrifolia* Kunth is called also as “Akar Kemeyan Paya” in Malaysia. These plants grow wild and are diffused particularly in Peninsular Malaysia. Sharlina *et al.* 2017 reported that the starch extracted from *D. pyrifolia* was characterized by the high amylose content (44.47 ± 1.86%); the same authors marked how *D. pyrifolia* Kunth represents a very important source of starch for food and non-food applications, such as crispy food, coating materials, hydrogels, films, bio-membranes, and adhesives (28).

*Dioscorea septemloba Thunb.*

Cholestane glycosides, dioseptemlosides A and B, together with six spirostane glycosides, dioseptemlosides C-H, were isolated from the rhizomes of *D. septemloba*. Among these, spirostane aglycones containing hydroxyl group at C-7 were reported in the family *Dioscoreaceae*. Spirostane-type glycosides showed strong activity on nitric oxide production (29). On the other hand, three new diarylheptanoids, dioscorol A, dioscorosides E_1_, E_2_; two new stilbenes, dioscorosides F_1_ and F_2_ were isolated from the rhizomes of *Dioscorea septemloba*. Besides, 1,7-bis(4-hydroxyphenyl)-hepta-4*E*,6*E*-dien-3-one, 1,7-bis(4-hydroxyphenyl)-1,4,6-heptatrien-3-one, 3,5-dihydroxy-1,7-bis(4-hydroxyphenyl)heptane, (3*R*,5*R*)-3,5-dihydroxy-1,7-bis(4-hydroxyphenyl)heptane 3-*O*-*β*-D-glucopyranoside, (3*R*,5*R*)-3,5-dihydroxy-1,7-bis(4-hydroxy-3-methoxyphenyl)-heptane 3-*O*-*β*-D-glucopyranoside, and 3-*O*-[*α*-L-arabinopyranosyl(1→6)-*β*-D-glucopyranosyl]oct-1-ene-3-ol were also obtained as known compounds. They have also shown a significant increase in the glucose consumption in differentiated L6 myotubes and displayed triglyceride inhibitory effects in HepG2 cells (Zhang *et al.*, 2017). Eight new compounds namely, dioscorosides G, H1, H2, dioscorol B, dioscorosides I, J, K1, and K2, together with twelve known ones were isolated from the rhizomes of *Dioscorea septemloba*. Moreover, the authors showed that some of these compounds were found to display significant inhibition of nitrite production evaluated *in-vitro *anti-inflammatory potential using LPS-stimulated RAW 264.7 murine macrophages (30). Currently, He *et al.* 2019 have identified and elucidated the structure of a new phenanthropyran, dioscorone B, and a new phenanthrene, together with seven known compounds, is from the 75% ethanol extract of *Dioscorea septemloba* rhizomes. Moreover, the authors reported that new isolated phenanthropyran, tested for antioxidant activity, showed excellent activities with IC_50 _values of 0.07 ± 0.10 μM and 0.13 ± 0.09 μM, respectively (31).

*Dioscorea tokoro Makino*

*D. tokoro *is one of those wild yams, and its rhizome was generally used for daily food.; in particular in the northern part of Japan, the rhizome was used as health-promoting food. *D. tokoro *was characterized by steroidal saponins, such as dioscin and gracillin, but no data were found about its health-promoting effect. The acetonitrile extract of *D. tokoro *rhizome fractionation led to protodioscin as an antiproliferative compound to HL-60 leukemic cells (32).

*Dioscorea villosa L.*

The most well-known species of this genus is *Dioscorea villosa*, also called “wild yam.” Two furanostane type saponins, namely methyl parvifloside, and protodeltonin, were isolated from *D. villosa* as well as two spirostane types deltonin and glucosidodeltonin (zingiberensis I), whereas minor saponins were methylprotodioscin, disoscin, and prosapogenin (33). The same authors reported that two flavan-3-ol glycosides were also isolated from *D. villosa *and fifteen steroidal compounds, including two new metabolites, were isolated from the tubers of *D. villosa*; these compounds were characterized as cholestane type steroidal glycosides named as *dioscoreavillosides A and B* (33). Dong *et al.*, 2012 described how the rhizomes were also afforded 14 diarylheptanoids, five of which were new compounds containing a tetrahydropyran ring in the heptane portion of the molecule (34). In another study, three furostanol saponins, parvifloside, methyl protodeltonin, and trigofoenoside A-1 were isolated from the *n*-butanol soluble extract of *D. villosa *by using HSCCC; moreover the authors reported that subsquent normal-phase HSCCC separation also led to the identification of four spirostanol saponins identified as zingiberensis saponin I, deltonin, dioscin, and prosapogenin A of dioscin (35). On the other hand, two series of lipidated steroid saponins were isolated from *D. villosa *(36). The series A was identified as a mixture of five lipidated steroid saponins: 5-en-spirostanol-2′-*O*rha-3-*O*-glucoside-6′-*O*-hexadecanoate, 5-en-spirostanol-2′-*O*-rha-3-*O*-glucoside-6′-*O-*octadecanoate, 5-en-spirostanol-2′-*O*-rha-3-*O*-glucoside-6′-*O*-9*Z*-octadecenoate, 5-enspirostanol- 2′-*O*-rha-3-*O*-glucoside-6′-*O*-9Z,12Z-octadecadienoate, and 5-en-spirostanol-2′-*O*-rha-3-*O*-glucoside-6′-*O*-9*Z*,12*Z*,15*Z*-octadecatrienoate. The series B was characterized as a mixture of the following five compounds: 5-en-clionasterol-3-*O*-glucoside-6′-*O*-hexadecanoate,5-enclionasterol-3-*O*-glucoside-6′-*O-*octadecanoate,5-en clionasterol-3-*O*-glucoside-6′-*O*-9*Z-*octadecenoate, 5-en-clionasterol-3-*O*-glucoside-6′-*O*-9*Z*,12*Z*-octadecadienoate, and 5-enclionasterol-3-*O*-glucoside-6′-*O*-9*Z*,12*Z*,15*Z*-octadecatrienoate (36). These findings revealed two classes as new biomarkers: the diarylheptanoids and the lipidated steroid saponins.

*Dioscorea zingiberensis C. H. Wright*

The rhizome of *Dioscorea zingiberensis* C.H. Wright is known as ‘‘Huang Jiang” and represents an important source of diosgenin. *D. zingiberensis *were used for the treatment of lung heat, pyretic stranguria, anthracia, coronary heart disease, and swelling; in particular, the rhizome of *Dioscorea zingiberensis* is extensively used for the extraction of diosgenin sapogenin and its glycoside dioscin, used for the synthesis of sex hormones and corticosteroids. It is worth mentioning the current study of Zhang *et al.*, 2018 that give comprehensive overview of the traditional usage and phytochemistry of the *Dioscorea zingiberensis *C.H. Wright: -more than 70 compounds have been identified; -several of these have been tested in preclinical assays and clinical trials; -a wide spectrum of biological effects including cardiovascular, anti-thrombosis, hyperlipidemia, neuroprotection, anti-inflammatory, and anthelmintic effect has been verified (37). Wang *et al.* 2010 reported how spirostanol based aglycon containing steroidal saponins are the representative steroidal saponins, and their quantity is higher than that of furostanol steroidal saponins in *D. zingiberensis *(38)*.* Apart from the steroidal saponins, other kinds of constituents were also identified from *D. zingiberensis*. Until now, four steroids have been isolated from this plant including *β*-sitosterol, sterol, zingiberenin F, and (25*R*)-3*β*-hydroxyspirost-*Δ*5,20(21)-dien-3-*O*-*β*-D-glucopyranosyl-(1→4)-[*α*-L-rhamnopyranosyl-(1→2)]-*β*-D- glucopyranoside. In addition to the common steroidal saponins, other constituents classified as alkaloid, phenyl-glycoside, and benzoic acid derivatives have also been determined. 4-aminomethyl-phenol, 2-*O*-*β*-D-glucopyranosyl-4-hydroxybenzoic acid and another nonsteroidal saponin called 2-*O-β*-D-glucopyranosyl-4-methoxybenzoic acid, were isolated and identified from the *n*-buthanol extracts of fresh rhizomes. Following the regular phytochemical research procedure, five compounds were obtained from *D. zingiberensis *(38). They consisted of two new phenanthrene derivatives, namely 2,5,7-trimethoxy-1,4-dione-9,10-dihydrophenanthrene and 2,5,6-trihydroxy-3,4-dimethoxy-9,10-dihydrophenanthrene; a new anthracenedione, *i.e.*, 2,5,7-trimethoxy-1,4-dione-anthracene; and two known 9,10-dihydrophenanthrenes, called 5,6-dihydroxy-2,4-dimethoxy-9,10-dihydrophenanthrene and 2,5-dihydroxy-3,4,6-trimethoxy-9,10-dihydrophenanthrene. One new bibenzyl (3,5-dihydroxy-4,40-dimethoxybibenzyl.) and one new phenanthrene (2,5-dihydroxy-4,6-dimethoxy-9,10-dihydrophenanthrene.), together with two known bibenzyls (3,5-dihydroxy-4-methoxybibenzyl 3,5′,-dihydroxy-3′,4-dimethoxybibenzyl and four known diarylheptanoids ((3*R*,5*R*)-3,5-dihydroxy-1,7-bis(4-hydroxyphenyl)heptane, (3*R*,5*R*)-3,5-dihydroxy-1-(3,4-dihydroxyphenyl)-7-(4-hydroxyphenyl)heptane, (3R,5*R*)-3,5-dihydroxy-1-(4-hydroxy-3-methoxyphenyl)-7-(4-hydroxyphenyl)-heptane and (3*R*,5*R*)-3,5-dihydroxy-1,7-bis(4-hydroxy-3-methoxyphenyl)-heptane were isolated from the dichloromethane soluble fraction of the rhizomes of *D. zingiberensis *(39)*.* All these phenolic compounds were evaluated for their anti-pancreatitic activities on sodium taurocholate-induced pancreatic acinar necrosis as inhibition of necrotic cell death pathway activation. Among them, (3*R*,5*R*)-3,5-dihydroxy-1-(3,4-dihydroxyphenyl)-7-(4-hydroxyphenyl) heptane which bears a trihydroxy-diarylheptane skeleton was shown to protect against pancreatic acinar cell injury through mediating novel potential therapy for pancreatic necrosis (39). 

**Biological activities** ***Dioscorea*** **plants**

The tubers of different *Dioscorea* species present a wide variation of bioactive compounds, responsible for their pharmacological activities. Generally, the evaluation of bioactive components and the assessments of their interactions could be viewed as the first step for the determination of potential of a plant (40-46). Also, their ethnopharmacological importance promotes further investigations of *Dioscorea* metabolites as a source of potential therapeutic agents to ameliorate different diseases (Table 1).

*In-vitro biological activities of Dioscorea species*

*Cytotoxic activity*

*D. bulbifera* ethanolic crude extract and different fractions (hexane, ethyl acetate, and water) at concentrations of 50, 100, and 200 μg/mL were found to possess a potent cytotoxic activity against human colorectal carcinoma (HCT116), human colorectal adenocarcinoma (HT-29), human lung carcinoma (A549), human breast carcinoma (MCF-7), human cervix epidermoid carcinoma (Ca Ski), and human colon normal (CCD-18Co) (60). The D. bulbifera ethyl acetate fraction (DBEAF), significantly inhibited the survivability of HCT116, HT-29, and A549 cells; particularly DBEAF had the best potency against HCT116 cells and reduced its cell viability in a time-dependent manner, having IC_50_ values of 163.59, 88.49, and 37.91 μg/mL at 24, 48, and 72 h, respectively and did not exhibit any cytotoxic effect towards the normal colon cells (CCD-18Co). DBEAF acts by increasing significantly the induction of early and late apoptosis, characterized by chromatin condensation, cell shrinkage, and DNA fragmentation as shown by flow cytometry. The authors also revealed that DBEAF induces apoptosis effects on HCT116 cells through externalization of phosphatidylserine and by promoting the loss of mitochondrial membrane potential (MMP), dysregulation of the Bcl-2 family proteins and the downregulation of the expression of procaspase -8, -9, -10 and -3 and PARP protein expression. Furthermore, their results suggested that the inhibitory effect of DBEAF on ERK1/2 and the activation of JNK were closely associated with the apoptotic cell death induction in human colorectal carcinoma (60). *D. collettii *var. hypoglauca and its active metabolite protoneodioscin, a furostanol saponin, was also evaluated for its cytotoxic activity against 60 cell lines including human leukemia, melanoma, and cancers of lung, colon, brain, ovary, renal, breast, and prostate. Protoneodioscin exhibited significant cytotoxicity with 50% growth inhibition (GI_50_) ranging from 0.5 to 9 mM against all cell lines from leukemia and most solid tumors (64). In the following year, the same research team evaluated two other compounds, namely; methyl protoneogracillin and gracillin. The results revealed that methyl protoneogracillin exhibited significant cytotoxicity with (GI_50_) ≤ 2 mM. Leukemia, CNS cancer, and prostate cancer were the most sensitive subpanels, while ovarian cancer was the least sensitive subpanels. On the other hand, gracillin lacked selectivity against human cancer diseases (29). A different approach was undertaken by Ashri and co-workers (2018) (71), who evaluated the cytotoxicity of modified *D. hispida* starch-based hydrogels on small intestine cell line (FHS-74 Int) to ensure the safety of their use. Their results revealed that the prepared hydrogels did not show any cytotoxicity and could be employed for future pharmaceutical use.

*Immunostimulant activity*

A mucopolysaccharide of yam (*D. batatas *Decne) (YMB) was found to increase the cytotoxic activity of mouse splenocyte against leukemia cell at 10 µg/mL concentration. However, it did not affect the viability of splenocytes. The production of IFN-γ was significantly increased in the YMP treated splenocytes. At 50 µg/mL concentration, it increases the up taking capacity and lysosomal phosphatase activity of peritoneal macrophages. In addition, YMP (10–100 µg/mL) significantly increased the viability of peritoneal macrophages (54). Dioscorin, the glycoprotein isolated from *D. alata* was reported to activate Toll-like receptor 4 (TLR4) and induce macrophage activation via typical TLR4-signaling pathways at 100 µg/mL concentration. It induces TLR4-downstream cytokine expression in bone marrow cells isolated from TLR4-functional C3H/HeN mice but not from TLR4-defective C3H/HeJ mice. Dioscorin also stimulated multiple signaling molecules (NF-jB, ERK, JNK, and p38) and induced the expression of cytokines (TNF-a, IL-1b, and IL-6) in murine RAW 264.7 macrophages (47).

*Neuroprotective activity*

Different *Dioscorea* species were used traditionally for the treatment of memory-related disorders and dementia, *i.e.* Alzheimer disease and other neurodegenerative diseases (82). The chloroform extract from *D. opposite *rhizomes significantly reduced the glutamate-induced toxicity in a dose-dependent manner with a maximum neuroprotective effect at 10 µg/mL (82). Kim *et al.*, 2011 reported the agonistic action of a 1:3 mixture of *D. japonica* Thunb. (DJ) and *D. nipponica* Makino (DN) rhizomes which contribute to their neuroprotective effect against diabetic peripheral neuropathy (16). Moreover, the compounds isolated from *D. nipponica* rhizomes exhibited anti-neuroinflammatory and neuroprotective activities, the most active of which was 3,7-dihydroxy-2,4,6- trimethoxy-phenanthrene. The later was the most potent nerve growth factor (NGF) inducer, with 162.36% stimulation, and strongly reduced nitric oxide (NO) levels with an IC_50_ value of 19.56 μM in LPS-activated BV2 microglial cells. Also, it significantly increased neurite outgrowth in mouse neuro2a (N2a) cells and therefore possessed a significant neuroprotective activity (76).

*Antidiabetic activity*

Different extraction procedures as ultrasonic-assisted extraction, cold water extraction, warm water extraction and hot water extraction significantly influenced the antidiabetic potential of the rhizomes of *D. hemsleyi *as determined via both alpha-glucosidase and alpha-amylase inhibition assays, where the IC_50_ values of alpha-glucosidase inhibition assay varied from 27.41 to 274.36 µg/mL while those of alpha-amylase inhibition assay varied from 3.66 to 47.57 mg/mL (70).

*Antioxidant activity*

The antioxidant activity of the extracts of *Dioscorea* species received much scientific attention (67, 72, 73 and 83). Different extracts of rhizomes of *D. hemsleyi, D. hispida *Dennst, *D. opposite* Thunb., *D. nipponica* Makino, *D. esculenta* (Lour). Burkill, *D. japonica* Thunb. var. pseudojaponica and *D. pentaphylla L. *were all studied for their antioxidant properties by different *in-vitro *assays namely; reducing power assay, ferric reducing antioxidant power assay (FRAP), DPPH radical scavenging assay, hydroxyl radical scavenging assay, superoxide radical assay, self-oxidation of 1,2,3-phentriol assay and antioxidant activity by radical cation (ABTS) assay. Generally, the studied extracts from different *Dioscorea* species showed a significant antioxidant potential which may account for their involvement in the treatment of various disorders via radical scavenging mechanisms.

*Anti-inflammatory activity*

Lipopolysaccharide-stimulated RAW 264.7 cells were employed to determine the anti-inflammatory properties of the ethanolic extract of tubers of *D. japonica* Thunb. var. *pseudojaponica *and individual steroid glycosides isolated from *D. septemloba* rhizomes (29, 73). *D. japonica* extract at doses of 500 and 1000 µg/mL significantly inhibited LPS-induced iNOS and COX-2 protein expressions, also, the nitrite relative concentration percentage ranged between 11.3% to 107.5% for the steroid glycosides isolated from *D. septemloba* rhizomes. Similar to the total ethanol extract of *D. membranacea* presented a potent inhibitory activity, with an IC_50_ value of 23.6 µg/mL. Its most potent metabolite included diosgenin-3-O-alpha-L-rhamnosyl (1-->2)-beta-D-glucopyranoside that showed a powerful inhibitory activity with IC_50_ value as low as 3.5 µg/mL (74). The study of the mechanism of anti-inflammatory activity of the ethanolic extract from the bark of *D. batatas* DECNE showed that it inhibited both NO and PGE2 production with an IC_50_ of 87–71 µg/mL respectively. It was also suggested that the extract act suppressing DNA-binding activity and reporter gene activity as well as translocation of the NF-jB p65 subunit. It also down-regulated IjB kinase (IKK), thus inhibiting LPS-induced both phosphorylation and the degradation of IjBa. In addition, it also inhibited the LPS-induced activation of ERK1/2 (55). A current study of Hwang *et al.* 2019 reported the anti-inflammatory effect of aerial bulblets of *Dioscorea* *japonica* Thunb extract through inhibition of NF-κB and MAPK signaling pathway in RAW 264.7.

*Antiallergic activity*

*D. membranacea* ethanolic extract and compounds showed antiallergic activities by inhibiting beta-hexosaminidase release as a marker of degranulation in RBL-2H3 cells, with an IC_50_ value of 37.5 µg/mL. From this extract, dioscorealide B showed the highest activity with an IC_50 _value of 5.7 µg/mL (77).

*Anti-pancreatitis and anti-arthritic activities*

Du *et al.* 2017 reported how several compounds were isolated from the rhizomes of *D. zingiberensis* *i.e.* (3R,5R)-3,5-dihydroxy-1-(3,4-dihydroxyphenyl) -7-(4-hydroxyphenyl) heptane at a concentration of 0.5 mM showed anti-pancreatitis activities on sodium taurocholate -induced pancreatic acinar necrosis with an inhibitory effect of 19.0%. Moreover, the same authors marked that this compound’s protective effects were mainly dependent on ATP inhibition and excessive ROS production and thus saving the cells from mitochondrial dysfunction (39). Another study reported that the steroidal saponins obtained from the rhizomes of *D. zingiberensis* showed anti-arthritic activities on the LPS stimulated 264.7 macrophage cells through induction of both p65 and IκBα protein expressions (98).

*Antibacterial activity*

The methanolic extract of tubers of *D. pentaphylla* L. showed antibacterial activity against *S. pyogenes, S. mutans, V. cholera, S. typhi,* and *S. flexneri*. The best activity was: 19.00 mm and 16.00 mm inhibition zone diameter observed (at 50 μg/disc and 200 μg/disc) against *S. pyogenes* using disc diffusion, agar well diffusion assays, respectively. The MIC values were 100 μg/mL for the extract against *V. cholera*, *S. typhi* and *S. flexneri* while it was 50 μg/mL against *S. pyogenes* and *S. mutans.* Similarly, Rajendra *et al.* reported the antibacterial activities of 5% and 10% methanolic extract of *D. deltoidea* Wall ex Griseb rhizomes against *S. aureus* and *E. coli,* but this concentration does not show activity against *S. typhi* and *P. aeruginosa*. It could be concluded that, in general, *Dioscorea* species do not exhibit a potent antibacterial activity (86).

*Antifungal activity*

The study of Cho *et al.* 2013 studied dioscin, isolated from wild yam *D. nipponica* root bark, for its potential as an antifungal agent via the propidium iodide assay and calcein-leakage measurement, in addition to, its ability to disrupt the plasma membrane potential, using 3,3′-dipropylthiadicarbocyanine iodide and bis-(1,3-dibarbituric acid)-trimethine oxanol. As reported by Cho *et al.* 2013 the results showed that dioscin exerts a considerable antifungal activity, where, the dye-stained cells showed a significant increase in fluorescent intensity after treatment with dioscin, demonstrating that dioscin possess an effect on the membrane potential. The auhors concluded that dioscin could act by disrupting the fungal membrane structure resulting in cell death (99).

*In-vivo studies on genus Dioscorea*

Globally a large number of *Dioscorea* plants have investigated *in-vivo*. Data on *Dioscorea* species potency on the animal model are summarized in Table 1.

*Animal models studies*

*The anti-constipation activity*

*Dioscorea opposita* Thunb. yam tuber is rich in starch, which has always been ignored and discarded during the isolation of bioactive compounds. Huang *et al.*, 2016 studied the anti-constipation effect of native yam starch (NS), dual enzyme-treated starch (DES), and cross-linked carboxymethyl starch (CCS) in constipation model induced by loperamide in KM mice. The modified starches (DES and CCS) could benefit the bowel function by increasing stool frequency and defecation moisture as they have the good water-binding capacity and swelling ability, the modified starches could increase the water-holding capacity of stools to remain soft. DES and CCS groups had improved small intestinal propulsion through the production of more short chain fatty acids (SCFAs) and decrease pH by increasing acetic acid and butyric acid concentration in the feces. The study done on mice with high-fat diet showed that both native and modified starch from Yam had anti-hyperlipidemic activity through a significant decrease of both cholesterol and triglycerides and the liver index. The reduction of superoxide dismutase (SOD) and increase malondialdehyde (MDA) was observed in hyperlipidemic mice while they were both improved in all starches-treated mice proving their antioxidant effect (Table 1) (100).

*Gastroprotective activity*

Four types of resistant starch (RS) including native (RS2), retrograded (RS3), cross-linked with sodium trimetaphosphate/sodium tripolyphosphate (RS4) and complexed with palmitic acid (RS5) resistant starches from winged yam (*D. alata* L.) showed the gastroprotective activity of in ethanol-induced gastric ulcer in mice. The increase in gastric emptying rate may help in reducing the contact time of ethanol and gastric mucosa and helps to prevent the gastric tissue damage. Therefore, an increase in gastric emptying induced by RS is a possible mechanism of anti-gastric ulcer. Moreover, RS3 and RS5 groups generated more short chain fatty acids which are produced through fermentation of the starch by the colon microflora. The fatty acids have many physiological and biological functions, such as increasing the immunity of the stomach. EtOH-ulcer can result from the imbalance between the generated reactive oxygen species (ROS) and the natural antioxidant power. Thus, the free radical scavenging is one of the mechanisms for inhibition of gastric ulcer (101). Mao *et al.* 2018 reported the decrease of SOD activity in ethanol-treated rats with an increase in MDA levels in gastric mucosa indicating severe oxidation reaction in ethanol-induced gastric ulcer mice. RS3 (6.4 g/kg) and RS4 (6.4 g/kg) exhibited the best antioxidant effect. Therefore, the inhibition of oxidative stress by the four types of RS is a possible mechanism that may assist in decreasing the gastric mucosal damage. Generally, the high dose groups of RS3 and RS5 (6.4 g/kg) showed the best activity (50). A current study also reported the protective effects of *Dioscorea batatas* flesh and peel extracts against an ethanol-induced gastric ulcer in mice (57).

*Anti-thrombotic activity*

Li *et al.* 2010 reported the significant anti-thrombotic and anticoagulation effect of the total steroidal saponin extract (TSSE) from the rhizomes of *D. zingiberensis* C.H. Wright on inferior vena cava ligation thrombosis rat model and pulmonary thrombosis mice model. Oral administration of the extract significantly inhibited adenosine diphosphate-induced platelet aggregation (PAG) *in-vivo*, which was more effective than *Xue-sai-tong*, a commercial product of triterpenoid saponins from *Panax ginseng* having antithrombosis activity in China. In addition, TSSE inhibited the thrombosis in a dose-dependent manner (more than 50% inhibition rate) which was more powerful than the standard Xue-sai-*tong* at 64.7, and 129.4 mg/kg doses of TSS, and markedly reduced thrombus weight. TSSE also exhibited in a dose-dependent manner prolongation of thromboplastin time (APTT) (which is an intrinsic clotting index), prothrombin time (PT) (that evaluates the extrinsic clotting pathway), and thrombin time (TT) (which is a test of fibrin formation, the addition of thrombin directly induces that). The more pronounced effect of TSSE on PT and TT than APTT indicates that it inhibits the extrinsic pathway of coagulation and fibrin formation, and cut down the thrombotic risk (102). 

*Anti-inflammatory activity*

The ethanolic extract of the tubers of *D. japonica* Thunb. var. *pseudojaponica* (EEDJ) showed *in-vivo *anti-inflammatory activity by significantly inhibiting the development of Carrageenan-induced paw edema in mice after the 4th and 5^th^ hour of the treatment at 1.0 g/kg. It also decreased the levels of TNF-α, which is a mediator of Carrageenan-induced inflammatory response and induced the release of kinins and leukotrienes. Carrageenan-induced paw edema causes oxidative stress with the release of peroxidation products, including malondialdehyde (MDA) and nitric oxide. MDA increased levels of oxygen free radicals, which attack polyunsaturated fatty acids in cell membranes and causing lipid peroxidation. EEDJ was able to reduce nitric oxide which was excessively released after administration of carrageenan and also could reduce the levels of MDA through increasing the enzymatic antioxidant defense systems such as catalase (CAT), superoxide dismutase (SOD) and glutathione peroxidase (GPx) in the paw oedema which are the natural protectors against lipid peroxidation (73).

The anti-arthritic effect of the total saponin extract from the rhizomes of *D. zingiberensis *C.H. Wright (TSRDZ) in Freund’s complete adjuvant (FCA) induced arthritis in rats (98). In this study, a significant increase in arthritis scores and ankle perimeter were observed after 4 days from intraplantar administration of FCA, which reaches its maximum on day 16. Administration of TSRDZ at 100 and 200 mg/kg produced substantial dose suppressive significant effect in the treated groups. TSRDZ at 100 and 200 mg/kg also caused a marked reduction of FCA-induced elevated indexes of the thymus and spleen. The high dose of TSRDZ caused only mild synovial infiltration with few inflammatory cells and no obvious damage in cartilage bone erosion, which appeared in histopathological study. The efficient regulation of TSRDZ on the inflammatory cytokines at 200 mg/kg, was almost equivalent to that caused by the standard methotrexate. Moreover, the mitigation of TSRDZ on the prostaglandins (PGE2), at 200 mg/kg, was almost equivalent to that of standard methotrexate. Furthermore, administration of TSRDZ at 200 mg/kg resulted in attenuating the production of MDA and NO, while elevating the SOD activity compared with the control group (98).

The total 80% ethanol extract of the tubercles of *D. trifida* L.f. as well as its three subfractions, dichloromethane, butanol, and aqueous fractions showed anti-inflammatory activity in food allergy induced by ovalbumin in BALB/c mice (94). Ova-sensitized BALB/c mice received the Ova solution to develop local signs of inflammation characterized by eosinophil infiltration, edema, an increase in a number of mast cells in the intestinal submucosa, mucus secretion, an increase in serum anti-OVA IgG1 and IgE, and weight loss because of hyper-catabolism caused by the production of inflammatory cytokines. The crude extract of *D. trifida* tested at the doses of 100 and 300 mg/kg/day corresponding to 36.6 and 109.8 mg/kg/day of allantoin, and 3.0 and 9.0 mg of diosgenin correlated substances/kg/day, respectively did not affect the body weight, but they inhibited IgE production compared to untreated groups. The inhibition tended to be more significant in aqueous fraction and butanol fraction, which contains allantoin, than dichloromethane, which contains diosgenin. The ethanolic extract and its fractions exerted a reduction in several mastocytes and edema, which was also probably due to the presence of diosgenin and allantoin (94). Diosgenin was also reported to reduce the production of anti-OVA IgE and the inflammatory infiltrate in allergic BALB/c mice intestines, resulting in a reduction in edema (103). BALB/c mice having food allergy showed an increase in mucus production by the caliciform cells in the small intestine (104). An important allergic response that is induced by interleukins IL-4 and IL-3 is hypersecretion of mucus. Mucus has a protective effect on the intestinal cells by limiting the antigen absorption. The presence of eosinophil peroxidase (EPO) indirectly reflects the infiltration of eosinophils into intestinal mucosa, which was reduced by administration of the ethanolic extract of *D. trifida*. Mollica *et al.* also showed that *D. trifida* extract and fractions reduced all of the inflammatory parameters associated with food allergies in Ova-sensitised animals and this activity is correlated with the presence of allantoin and diosgenin correlated substances (94). The animals treated with ethanolic extract showed a reduction in mucus production, and this result was also observed for the other fractions. Previous studies showed that diosgenin and allantoin activities reduce mucus production in animals allergic to OVA (103).

The crude extracts of *D. membranaceae* and *D. birmania*, which contain high amounts of sapogenins, inhibited the production of nitric oxide (NO) most probably through inhibiting the nitric oxide synthetase (iNOS) and TNF-α, with consequent reduction of intestinal edema (74, 77). A study by Olayemi and Ajaiyeoba also associated the antiedematogenic activity of crude extract from *D. esculenta* to the presence of sapogenins (105). *In-vivo *studies have demonstrated that oral administration of allantoin attenuates the production of IgE, IL-4, and IL-5, has an anti-inflammatory effect and promotes healing (68).

The methanol extract of the rhizomes of *D. deltoidea* Wall.ex Griseb. at 200 mg/kg inhibited the rat hind paw carrageenan-induced edema. The maximum percentage of inhibition was achieved at 3 h after the intraperitoneal administration of the extract (106).

*Neuroprotective activity*

In a study made by Yang *et al.*, the chloroform soluble extract of the rhizomes of *D. opposita *(CSDO) showed cognitive enhancing effects on spatial memory and learning function of mice against scopolamine-induced amnesic deficits via Morris water maze and passive avoidance tests. In Morris water maze test, both acute treatment by CSDO (200 mg/kg body weight, p.o.) and prolonged-treatment (10 days’ daily administration of 50 mg/kg body weight, p.o.) exhibited significant shorter escape latencies in daily first trial than the scopolamine-administered group during a 4-consecutive-day training period, which suggested that CSDO improves the impaired reference memory (long-term memory) induced by scopolamine. Especially, the acute treatment group showed a marked decrease in escape latencies in each daily trial compared with the prolonged treatment group. In this study, CSDO also showed significant enhancing effects on spatial memory retention in the probe trial. Similar results were obtained in the passive avoidance test. CSDO treatment significantly increased step-through latencies of scopolamine-induced amnesic mice compared to control untreated group. The results of the two animal behavioral tests demonstrated that CSDO improves spatial learning and memory function against scopolamine-induced amnesia (82). The study of Jeon *et al.*, 2014 investigated the neuroprotective effect of herbal mixture from *Euphoria longana*, *Houttuynia cordata,* and *Dioscorea japonica*, by testing the hypothesis that administration of herbs reverses memory deficits and promotes the protein expression of brain-derived neurotrophic factor (BDNF) in the mouse brain: these herbs demonstrated an inductive effect on BDNF, cyclic-AMP response element-binding protein (CREB) and phospho-CREB (pCREB) (107).

*Antinociceptive activity*

The study of the antinociceptive effect of the methanol extract of the bulbs of *Dioscorea bulbifera* L. var *sativa* (MEDB) was performed by Nguelefack *et al.* (2010) The effects of MEDB persisted for 5 days after two administrations in CFA-induced hypernociception at 250 and 500 mg/kg. MEDB significantly inhibited acute lipopolysaccharides (LPS)-induced pain at 500 mg/kg but did not affect thermal hypernociception and capsaicin-induced spontaneous nociception. The antinociceptive effects of MEDB in prostaglandin-E2 (PGE2) model was antagonized by either nitro-L-arginine methyl ester (l-NAME) or glibenclamide. This indicated that the plant exerted its antinociceptive effect through partial activation of the NO–cGMP–ATP-sensitive potassium channels pathway (62).

*Antihyperlipidemic and antioxidative activity*

The intraperitoneal administration of n-butanol extract of the *D. nipponica* rhizomes at 50 mg/kg regulates the blood cholesterol, triglyceride, HDL and LDL than the chloroform (50 mg/kg) and water extracts (50 mg/kg) (80). Wang *et al.* 2012 reported the potential activity of trillin, a steroidal saponin isolated from *D. nipponica* rhizomes, as anti-hyperlipidemic and anti-oxidative agent *in-vivo*. The intraperitoneal administration of trillin (0.5 mg/kg dissolved in 0.5% DMSO) enhanced the blood circulation and could increase the bleeding time for more than 50% and coagulation in the rats fed on a high-fat diet in which the blood viscosity would be changed resulting in shorter blood coagulation time. The improvement effect of trillin in restoring the blood coagulation abnormality in high-fat diet fed rat was due to its ability to reduce the levels of blood cholesterol, triglyceride and two critical lipoproteins (HDL and LDL). The level of lipid oxidation was increased in high-fat diet rats, causing oxidative stress. Trillin exerted an anti-oxidative effect through lowering lipid peroxidation, via the enhancement of superoxide dismutase (SOD) level in blood. Thus, the combination of trillin and lovastatin in treating hyperlipidemia could represent a more effective therapy (Wang *et al.* 2012). The authors of this research marked how these findings greatly support the significant role of *D. nipponica* in protecting the cardiovascular system *in-vivo *against hyperlipidemia (80). Tang *et al.* 2015 studied the mechanism by which the total saponins of the rhizomes of *D. nipponica* Makino (DN), *D. panthaica* Prain et Burkill (DP), and *D. zingiberensis* C.H. Wright (DZ), attenuated myocardial ischemia (MI) induced by injection of isoprenaline (ISO) in rats, and compared the therapeutic effect of total saponins from the three species on myocardial antioxidant levels and myocardial histology (87); the authors reported how high serum levels of lactate dehydrogenase (LDH), aspartate aminotransferase (AST) and creatine kinase (CK) indicated cell membrane damage and were thus elevated in the ISO model group. Intra-gastric injection of total saponins of the three species could restore the activities of myocardial injury marker enzymes to the same extent. Oxidative stress plays an essential role in the pathogenesis of MI injury. SOD, catalase (CAT), GPx, and total antioxidant capacity (T-AOC) levels were reduced significantly in the ISO model group with the increase in MDA. The activities of these enzymes were normalized by intragastric injection of the total saponins of the three species. The study revealed that the anti-MI mechanism of *Dioscorea* saponins is related not only to the enzymatic antioxidant, such as GPx and CAT but also to nonenzymatic antioxidants and its effect on myocardial histology (87). The current studies reported the beneficial effects of purple yam (*Dioscorea alata* L.) resistant starch on hyperlipidemia in high-fat-fed hamsters (108, 109).

*Uricosuric effect*

The total saponins from *Rhizoma Dioscoreae* Nipponicae could effectively reverse potassium oxonate-induced alterations in renal mouse urate transporter 1, glucose transporter 9, organic anion transporter 1, and organic anion transporter 3mRNA and protein levels, resulting in enhancement of renal urate excretion in mice (79). Moreover, the authors concluded that the total saponins from Rhizoma *Dioscoreae Nipponicae* had a uricosuric effect on the regulation of renal organic ion transporters in hyperuricemic animals. In a further paper, the same authors here was the potent uricosuric effect of TDN on hyperuricemic rats by decreasing the expressions of renal rURAT1 while increasing the expressions of renal rOAT1 and rOAT3 (78).

*Clinical trials*

A double-blind, placebo-controlled, cross-over study was conducted to evaluate the effects of a wild yam cream in 23 healthy women suffering from troublesome symptoms of the menopause. Every female was treated with the active cream and a placebo for 3 months randomly. After 3 months treatment with topical wild yam extract in women suffering from menopausal symptoms it was found that the cream is free from side-effects, and has little effect on menopausal symptoms (110). 

**Table 1:** Pharmacological activities of *Dioscorea* species

**Botanical name**	**Plant parts**	**Pharmacological activities**	
		***In-vitro***	***In-vivo***
*Dioscorea alata* L.	Tuber	Immune-modulating activity (47) Anti-inflammatory properties (48) Antibacterial activity (49)	Gastroprotective activity (50) Cardioprotective properties (51) Anti-inflammatory activity (52)
*Dioscorea antaly* Jum. and H. Perrier	Tuber	Toxicity on embryo-larval development (53)	Not known
*Dioscorea belophylla* (Prain) Haines	Tuber	Not known	Not known
*Dioscorea batatas *Decne	Tuber	Immune-modulating activity (54). Anti-inflammatory activity (55) Anti-severe acute respiratory syndrome associated coronavirus (56). Gastroprotective activity (57)	Enhance murine splenocyte proliferation *ex-vivo* and improve regeneration of bone marrow cells (58) Immunomodulatory activity (59) Anti-inflammatory activity (55).
*Dioscorea bulbifera *L.	Tuber, Rhizome	Anticancer/antitumor (60). Bacterial and fungal infections, Inflammation and pain, Diabetes and digestive problems, Oxidative stress and degenerative diseases (61).	Antinociceptive activity (62). Inflammation and pain, Diabetes and digestive problems, Tumor and cancer (61)
*Dioscorea cayenensis* Lam.-Holl	Tuber	Antifungal activity (12)	Not known
*Dioscorea collettii *var. hypoglauca	Rhizome	Anticancer activity (63, 64)	Not known
*Dioscorea deltoidea *Wall.	Rhizome	Antibacterial activity (65)	Anti-inflammatory activity (66)
*Dioscorea esculenta* (Lour.) Brukill	Tuber	Antioxidant activity (67)	Anti-inflammatory activity (68)
*Dioscorea hamiltonii *Hook.f.	Tuber	Antimicrobial activity (69)	Not known
*Dioscorea hemsleyi*	Rhizome	Antidiabetic activity and Antioxidant activity (70)	Not known
*Dioscorea hispida *Dennst.	Tuber, tendrils	No toxicity on small intestine cells (71) Antioxidant activity (72)	Not known
*Dioscorea japonica* Thunb. (DJ)	Rhizome	Neuroprotective activity (16) Antioxidant activity (73) Anti-inflammatory activity (73)	Anti-inflammatory activity (73)
*Dioscorea membranacea*	Rhizome	Anti-inflammatory activity (74) Immunomodulatory activity (75) Anticancer activity (17, 75).	Anti-inflammatory activity (74)
*Dioscorea nipponica *Makino	Tuber, rhizome	Neuroprotective activity (16, 76) Anti-neuroinflammatory activities (76) Antiallergic activity (77) Antifungal activity (Rajendra *et al.*, 2014) Uricosuric effect (78, 79)	Anti-hyperlipidemic (80) and antioxidative activity (81)
*Dioscorea oppositifolia *L. syn: *Dioscorea opposita *Thunb	Tuber Rhizome	Neuroprotective activity (82) Antioxidant activity (83) Mediated Synthesis of Gold and Silver Nanoparticles with Catalytic Activity (84)	Neuroprotective activity (82) Antidiabetic activity (85)
*Dioscorea panthaica* Prain et Burkill (DP)	Rhizome	Antioxidant activity (86) Antitumor activity (81) Anti-hypercholesterolemic effect (87) Anti-platelet aggregation activity (87) Antifungal activity (88)	Antihyperlipidemic and antioxidative activity (81)
*Dioscorea pentaphylla *L.	Tuber	Antioxidant activity and antibacterial activity (86)	Not known
*Dioscorea polygonoides *Humb. et Bonpl.	Tuber	Not known	Anti-hypercholesterolemia activity (89) Antidiabetes activity (90)
*Dioscorea preussii* Pax	Tuber	Antileishmanial activity (27) Antifungal activity (27)	Not known
*Dioscorea pubera *Blume	Tuber	Not known	Not known
*Dioscorea septemloba*	Rhizome	Anti-inflammatory activity (74) (91) Triglyceride inhibitory effects (31)	Anti-hyperuricemia activity (52)
*Dioscorea tokoro *Makino	Rhizome	Antiproliferative activity (32)	Anti-hyperuricemic activity (92)
*Dioscorea trinervia *Roxb.	Tuber	Not known	Not known
*Dioscorea trifida* L.f.	Tuber	Antioxidant activity (93)	Anti-inflammatory activity (94)
*Dioscorea villosa* Willd.	Tuber, root	Antibacterial activity (95) Hepatoprotective activity (96)	Antinociceptive activity (97) Anti-inflammatory activity (97) No acute or subchronic toxicity (97)
*Dioscorea zingiberensis* C. H. Wright	Rhizome	Anti-pancreatitis activity (39) Anti-arthritic activity (98) Anthelmintic activity and low toxicity (38) (Wang *et al.*, 2010)	Anti-arthritic effect, antioxidant and anti-inflammatory activities in rats (98) Antihyperlipidemic and antioxidative activity (81)

## Conclusion

*Dioscorea* species make a significant contribution both as root crops and vegetables to the diets around the world. Despite their importance as a food source, *Dioscorea* plant parts are quite useful in the treatment of various ailments and disorders due to the presence of several bioactive compounds such as diosgenin. However, several reported ethnomedicinal potential need to be validated and detailed investigations on *Dioscorea* pharmacological properties and phytochemical composition should be carried out to standardize the formulations used. Indeed, some pharmacological properties such as hypolipidemic, hypoglycaemic, antioxidant, anti-inflammatory, antimicrobial, antiproliferative, androgenic, estrogenic, and contraceptive have been reported. However, most of the active constituents have not been characterized. Thus, authentication of all the secondary metabolites (alkaloids, saponin, flavonoids, tannins, and phenols) from *Dioscorea* should be performed thoughtfully to standardize and validate its quality and biological potentials. Moreover, investigations are warranted to address the poor conversion of the preclinical results to clinical efficacity. Therefore, an attempt should be made to understand their mechanism of action, pharmacokinetics/pharmacodynamics, and bioavailability and to conduct clinical trials. Overall, these findings suggested that *Dioscorea* should not be ignored and should rather be considered as a good alternative source of active molecules that can prevent or alleviate both functional and infectious disease burden, representing a current big challenge in developing countries. The knowledge gaps in this review such as insufficient data on a clinical trial to support preclinical results will help the further initiative to turn *Dioscorea* into drug and stimulate activity to promote their production and utilization as not only valuable components of a well-balanced diet but also for disease prevention.
